# The Mediating Roles of Mental Health and Substance Use on Suicidal Behavior Among Undergraduate Students With ADHD

**DOI:** 10.1177/10870547221075844

**Published:** 2022-02-04

**Authors:** Natasha Brown, Margaret McLafferty, Siobhan M. O’Neill, Rachel McHugh, Caoimhe Ward, Louise McBride, John Brady, Anthony J. Bjourson, Colum P. Walsh, Elaine K. Murray

**Affiliations:** 1 Letterkenny Institute of Technology, Co. Donegal, Ireland; 2 Northern Ireland Centre for Stratified Medicine, School of Biomedical Sciences, C-TRIC, Altnagelvin Hospital, Ulster University, Derry/Londonderry, UK; 3 School of Psychology, Coleraine Campus, Ulster University, Coleraine, Co. Derry, UK; 4 Western Health and Social Care Trust, Tyrone and Fermanagh Hospital, Omagh, Co. Tyrone, UK; 5 Genomics Medicine Research Group, School of Biomedical Sciences, Coleraine Campus, Ulster University, Coleraine, Co. Derry, UK

**Keywords:** depression, substance use, college students, ADHD, suicide

## Abstract

**Objective:**

To evaluate the prevalence of suicidal ideation (SI), plans and attempts, and non-suicidal self-injury (NSSI) among students with attention deficit hyperactivity disorder (ADHD). Furthermore, we explored the mediating effects of depression, anxiety, alcohol and substance use on the association between ADHD and suicidal behaviors and NSSI.

**Method:**

Participants were first-year undergraduate students (*n* = 1,829) recruited as part of the World Mental Health International College Student Initiative. Participants completed validated clinical measures online.

**Results:**

The prevalence of suicide behaviors and NSSI were significantly higher among students with ADHD than those without. Mediation analyses indicated that ADHD directly and indirectly increased suicidal behaviors and NSSI. While ADHD increased suicidal behaviors and NSSI through depression, ADHD and the co-variates age and gender also had indirect effects on suicidal behaviors via substance use.

**Conclusions:**

Specific predictors of risk were identified for students with ADHD which may inform the development of more targeted mental health and suicide prevention strategies across campuses.

The mental health of university students is a matter of concern globally, with a growing body of literature highlighting this group as particularly susceptible to psychopathology (Sheldon et al., 2021). The World Mental Health International College Student Initiative (WMH-ICS) reported that over one third of college students screened positive for at least one clinically significant lifetime disorder, while 31% screened positive for at least one 12-month disorder (Auerbach et al., 2018). Moreover, high rates of suicide behaviors, including suicide ideation (SI), planning and attempts, and non-suicidal self-injury (NSSI), remain prevalent within this population (Eskin et al., 2016). Indeed, a recent meta-analysis revealed that 22% of US college students experienced SI, while 3% had made a suicide attempt (Mortier et al., 2018). Likewise, in Northern Ireland (NI) almost 20% of student participants engaged in NSSI, one fifth had made a suicide plan and 31% experienced SI (McLafferty et al., 2017; O’Neill et al., 2018).

For many young people the transition into adulthood coincides with the commencement of university which can be a highly stressful experience characterized by major psychosocial change. High levels of stress have been associated with first-onset psychopathology and the aggravation of pre-existing mental illness in undergraduate students (Bewick et al., 2010; Eisenberg et al., 2007). However, for students with a neurodevelopmental disorder, such as ADHD, the associated symptomology may exacerbate the stress incurred by university transition, thus increasing susceptibility to poor mental wellbeing.

There is a scarcity of literature concerning ADHD prevalence within post-secondary institutions. Preliminary estimates suggest rates range from 2% to 8% (DuPaul et al., 2009). However, a recent multinational study spanning nine countries reported a significantly higher prevalence, with 16% of students screening positively for ADHD. Moreover, the prevalence of ADHD differed cross-nationally, with rates as high as 21% and 27.7% reported in Northern Irish and Australian student samples (Mak et al., 2021). Furthermore, studies in Kenya and Iran have suggested ADHD prevalence rates of 22% and 25% respectively (Atwoli et al., 2010; Bakhshani et al., 2012). ADHD is characterized by impulsivity, inattention, and hyperactivity and is often accompanied by deficits in executive function and emotional regulation (American Psychiatric Association, 2013). Adults with ADHD frequently experience significant functional impairment and difficulties with relationships, organization, time, and stress management. Indeed, some of the major stressors associated with the university transition include navigating a less structured learning environment, establishing new social support networks and increased academic and self-regulatory demands. Consequently, adjustment to university life can be particularly challenging for those with ADHD (Fleming & McMahon, 2012).

Students with ADHD experience higher rates of mental illness (Fleming & McMahon, 2012; Mochrie et al., 2020). Findings indicate that between 54% and 77% of those with ADHD experience at least one comorbid disorder (Anker et al., 2018; Sobanski et al., 2007). The most common disorders include mood and anxiety disorders as well as substance and alcohol use disorders, which are particularly prevalent among young adult males (Anker et al., 2018; Piñeiro-Dieguez et al., 2016; Sobanski et al., 2007). Although increased suicide risk has been established within the general population, there remains limited research exploring the prevalence of suicidal behaviors among college students with ADHD (Barbaresi et al., 2013; Impey & Heun, 2012). Preliminary research suggests SI and suicide attempts to be three and four times higher, respectively, among students with ADHD (Eddy et al., 2020; Van Eck et al., 2015). Interestingly, recent findings imply no significant difference in the prevalence of suicide plans between ADHD and non-ADHD samples which may be attributable to higher levels of impulsivity associated with ADHD (Eddy et al., 2020).

Despite the high prevalence of ADHD and worrisome findings regarding mental health and suicidality, only a small body of literature examining the relationship between ADHD and suicidality among students exists. Subsequently, the mechanisms which underlie the association between ADHD and suicidality remain unclear (Septier et al., 2019). Some researchers propose that ADHD symptomology, such as impulsivity, inattention, and executive dysfunction present independent risk. For example, it was found that individuals who demonstrated high levels of both traits were more likely to have made a lifetime suicide attempt (Keilp et al., 2013; Wang et al., 2014). Additionally, McGirr et al. (2007) found that impulsivity was a significant predictor of suicide completion, more so than the presence of psychopathology. Direct links between ADHD and suicidality have been reported and even after adjusting for the presence of co-morbid disorders, individuals with ADHD demonstrated an increased risk of suicide (Chen et al., 2019; Eddy et al., 2020; Ljung et al., 2014). Nevertheless, alternative findings imply that this association is in fact entirely mediated by the presence of co-morbid disorders, particularly mood and anxiety disorders (Arsandaux et al., 2021; Balazs et al., 2014; Zhong et al., 2021). Indeed, associations between depression and suicidality have been well established and mood and anxiety disorders are some of the strongest predictors of suicidal behavior in young adults (Boden et al., 2007; Wang et al., 2019).

The limited pool of existing literature therefore presents mixed findings as to whether ADHD is a distinct risk for suicidality or whether the relationship is attributable to the presence of co-morbid disorders. Previous studies have focused on North American college students and rely on smaller samples not exceeding 904 students (Eddy et al., 2020; Van Eck et al., 2015; Zhong et al., 2021). Furthermore, many previous studies explore only the relationship between ADHD and SI. This too is a limitation, as the prevalence and predictors of suicidal ideation, plans and attempts, as well as NSSI often differ, where plans and attempts are stronger predictors of future suicide risk (Eddy et al., 2020; Nock et al., 2013). Additionally, due to the proposed impulsive nature of suicidality in those with ADHD, greater insight regarding the prevalence and predictors of planning is required (Impey & Heun, 2012). Lastly, despite the high prevalence of substance and alcohol use among students with ADHD and the associated increased suicide risk, there has been little focus on their potential mediating effects (Anker et al., 2018). Due to the increased accessibility of higher education, a growing number of students with ADHD attend universities (Sedgwick, 2018). Therefore, it is crucial to gain a better understanding of the mechanisms that underlie suicidality within this vulnerable population to ensure appropriate and effective supports are available.

The present study aims to address the above concerns and provide further insight into the associations between ADHD and suicidality. The prevalence of SI, plans and attempts as well as NSSI will be explored. Additionally, the relationship between ADHD and suicide behaviors, and ADHD and NSSI, will be examined. Finally, we will account for the mediating impact of depression, anxiety, substance and alcohol use, while controlling for age and gender variations.

## Methodology

### Design

The Student Psychological Intervention Trial (SPIT), which was conducted as part of the WMH-ICS, aims to gather information about student mental health and monitor this as students’ progress through university. The current study utilized data collected in September 2019 when undergraduate students first commenced college in Letterkenny Institute of Technology (LYIT) in the Republic of Ireland (ROI) and across the four Ulster University (UU) campuses in NI. The study was granted ethical approval from the Ulster University Research Ethics Committee (REC/19/0072).

### Participants

One week before registration, all first-year undergraduate students commencing degree courses at UU and a subset of those registering at LYIT were invited to partake in the study via email. The invitation email contained a participant information sheet outlining the study’s aims and methods. Following this, trained researchers and volunteers recruited students on the NI and ROI campuses during welcome meetings and after the students had registered for their course on campus. Students were provided with a link to the survey and a unique participant code. Overall 1,829 first-year students completed the online survey, and each received a university branded sweatshirt. The sample consisted of mainly Ulster University students (*n* = 1,469) and respondents were more likely to be female (*n* = 1,317) and under the age of 21 (*n* = 1,418). All participants were first-year undergraduate students, over the age of 18 and were residents of either NI or the ROI. Consistent with the exclusion criteria of other WMH-ICS studies, students under the age of 18, those repeating first-year and international students could not participate.

### Measures

The comprehensive online survey was completed online via Qualtrics software. The survey questions were adapted from the WMH-Composite International Diagnostic Interview Screening Scales (WMH-CIDI -SC) (Kessler & Ustün, 2004). The survey examines the prevalence of several mental health disorders, suicide behaviors and ADHD, in accordance with DSM-IV criteria.

#### ADHD

ADHD symptoms were screened for using the World Health Organization’s (WHO) standardized Adult ADHD Self-Report Scale (ASRS-v1.1). The six items are consistent with the DSM-IV criteria for adult ADHD and measure the frequency of ADHD symptoms in the previous 6 months. Responses were measured on a five-point scale with options ranging from *never* to *very often*. Total scores range from 0 to 6, with scores of four or more indicating probable ADHD. The measure has demonstrated high internal consistency as well as strong reliability and validity in adult samples (Adler et al., 2006; Matza et al., 2011). Moreover, the ASRS-v1.1 screener has shown good clinical validity and good concordance with blinded clinical diagnoses when compared to diagnostic interviews for adult ADHD. The screener has demonstrated a sensitivity of 68.7%, specificity of 99.5%, and classification accuracy of 97.9% (Kessler et al., 2005). In the current study the Cronbach’s α = .81.

#### Depression

The Patient Health Questionnaire (PHQ-9) was used to screen for depression (Kroenke & Spitzer, 2002). The PHQ-9 consists of nine items and responses are scored on a 0 to 3 scale. The questions relate to symptoms experienced during the previous 2 weeks. The current study utilized continuous scores and higher scores indicated more severe depression. The PHQ-9 has demonstrated strong reliability and validity as well as good psychometric properties (Wittkampf et al., 2007). In the current study the Cronbach’s α = .91.

#### Anxiety

The GAD-7 was used to screen for generalized anxiety disorder (Spitzer et al., 2006). This brief measure consists of seven items scored on a 0 to 3 scale. The questions relate to symptoms experienced during the previous 2 weeks. In the current study continuous scores were used and higher scores indicated more severe generalized anxiety. The GAD-7 has good reliability and factorial validity with Cronbach’s coefficient ranging from .85 to .92 (Spitzer et al., 2006): in the current study the Cronbach’s α = .96.

#### Alcohol use

The Alcohol Use Disorders Identification Test (AUDIT) was used to screen for problematic alcohol use (Saunders et al., 1993). The measure consists of 10 items that are designed to assess three domains: alcohol consumption, alcohol dependence, and problems caused by alcohol use. For the current study the three domain scores were summed to create a total AUDIT score. Higher scores indicated more hazardous alcohol use. The AUDIT has been validated with college students and demonstrates strong reliability (Kokotailo et al., 2004), with a Cronbach’s α = .82 in the current study.

#### Substance use

The WHO’s Alcohol, Smoking and Substance Involvement Screening Test (ASSIST) was used to screen for substance use. The ASSIST is a brief measure in which participants indicate lifetime frequency of use of various substances, as well as social and functional impairments associated with usage. The six-point scale ranges from *every or nearly every day* to *never*. In the current study, higher scores indicated more problematic substance use. ASSIST has been found to be a reliable and valid screening test for drug use (Humeniuk et al., 2008). In the current study the Cronbach’s α = .95.

#### Suicidal behavior

The self-report version of the Self Injurious Thoughts and Behavior Interview (SITBI) was used to identify students with a lifetime history of SI, plans and attempts, and NSSI (Nock et al., 2007). If students reported that they had attempted suicide or made a suicide plan in the previous year, or that they may act on plans in the future, a high-risk alert was activated. These students were sent an email with signposting information and were subsequently contacted by Student Wellbeing (UU) or an accredited counselor (LYIT) via telephone.

#### Data analyses

Weights were created based on the gender and age characteristics of the first-year student population at UU and LYIT. The weights were applied to the data during analysis to ensure that the results were representative of the total student population. Mplus v.7.31 (Muthén & Muthén, 2017) was used for mediation analyses while SPSS v.26 was utilized for the remaining analyses. Missing values were dealt with using the full information maximum likelihood method in Mplus v.7.31.

The mediation analyses were conducted in three different stages as outlined below.

**Figure 1.**
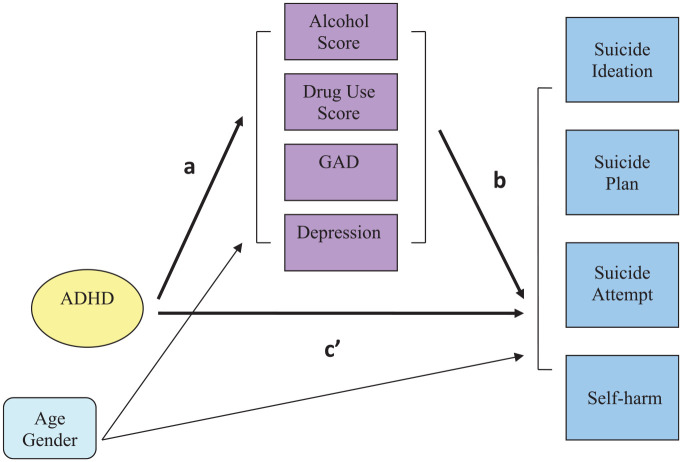
Multiple mediator model of direct and indirect effects.

1. Regression models estimated the direct effects between individuals with/without ADHD and the dichotomous dependent variables; lifetime SI, plans and attempts, and lifetime NSSI. The pathways of the covariates, gender and age, and the mediators (alcohol use, substance use, anxiety and depression scores) were fixed to zero.
2. The covariates, gender, and age were added to the model and the direct effects were estimated. The pathways to and from the mediators remained fixed to zero.
3. The pathways to and from the mediators were freed. Direct effects and indirect effects of ADHD and the covariates through the mediators were estimated. Direct pathways from ADHD to the mediators were also estimated. The mediation model is depicted in Figure 1.

### Results

#### Prevalence of suicide behaviors

Overall, there was a high prevalence of ADHD (27.2%), with 31.8% screening positive at LYIT and 26.1% at UU. The highest lifetime prevalence rates in all students were for SI (28%), suicide plan (14.3%), and NSSI (13.4%). In a comparison between students with and without ADHD, students with ADHD had rates of suicidal ideation that were more than twice as high (44.8% vs. 21.7%), suicide plans which were almost three times higher (26.6% vs. 9.8%) and both attempts (13.4% vs. 5.5%) and NSSI (23.4% vs. 9.6%) that were almost 2.5 times greater than students without ADHD (Table 1).

**Table 1.** Comparison of Prevalence of Suicide Behaviors Between Students With and Without ADHD.

Total	Total		Non-ADHD		ADHD		
	*n*		*n*	%	*n*	%	
	1,829		1,327	72.8	502	27.2	
Suicide behaviors	*n*	%	*n*	%	*n*	%	*X* ^2^
Suicide ideation	505	28	276	21.7	229	44.8	95.656*
Suicide plan	253	14.3	119	9.8	134	26.6	82.738*
Suicide attempt	131	7.7	64	5.5	67	13.4	31.742*
Non-suicidal self-injury	259	13.4	131	9.6	128	23.4	59.866*

*Note. n* = raw unweighted values, % weighted values.

* *p* < .001.

#### Mediation analysis

A range of model fit indices were assessed to determine the adequacy of the mediation models (Table 2). These indices included AIC (Akaike Information Criterion), BIC (Bayesian Information Criterion), and SSABIC (sample size adjusted BIC). Table 2 shows that the AIC, BIC, and SSABIC were lowest for model 3. Chi-square tests were also conducted using log-likelihood values and scaling correction factors obtained from the MLR estimation in order to help determine the best fitting model, with a significant difference revealed between models (*p* < .0001). Model 3 was determined to be significantly superior.

**Table 2.** Fit Indices Among Mediation Models.

Model	Log-likelihood	# Free parameters	AIC	BIC	SSABIC
Model 1	−24,198.579	16	48,429.158	48,517.343	48,466.511
Model 2	−24,167.084	24	48,382.167	48,514.444	48,438.197
Model 3	−23,803.062	52	47,710.123	47,996.723	47,831.521

*Note.* AIC = Akaike information criterion; BIC = Bayesian information criterion; SSABIC = sample size adjusted BIC.

##### Stage 1

The direct effects between the independent variable ADHD and the dependent variables were all significant as shown in Tables 3 to 6. The odds of individuals with ADHD endorsing SI, suicide attempt, and NSSI were almost three times higher and odds of endorsement of a lifetime suicide plan were almost three and a half times higher.

**Table 3.** Direct and Indirect Effects of ADHD on Suicide Ideation via Mental Health and Substance Problems With Co-Variates Gender and Age.

Variable	Direct effects			Indirect effects			
	Stage 1 *OR* (95% CI)	Stage 2 *OR* (95% CI)	Stage 3 *OR* (95% CI)	Alcohol scores ß (*SE*)	Drug use scores ß (*SE*)	GAD ß (*SE*)	Depression ß (*SE*)
ADHD (none)	2.925*** (2.302–3.718)	2.958*** (2.324–3.766)	2.075*** (1.589–2.708)	.036 (0.025)	.077 (0.027)**	−.007 (0.027)	.297 (0.057)***
Gender (male)		0.960 (0.752–1.226)	1.000 (0.772–1.297)	−.016 (0.012)	−.052 (0.018)**	−.003 (0.011)	.032 (0.023)
Age (under 21)		1.594** (1.221–2.082)	1.592** (1.200–2.111)	−.016 (0.012)	.039 (0.018)*	.000 (0.002)	.001 (0.024)
Alcohol scores			1.016 (0.994–1.039)				
Drug use scores			1.107* (1.044–1.173)				
GAD			0.997 (0.978–1.017)				
Depression			1.081*** (1.055–1.109)				

*Note. OR* = odds ratio; CI = confidence interval; ß = beta coefficient; *SE* = standard error. All significant values are presented in bold.

*** *p* < .001. **p < .01. *p < .05.

**Table 4.** Direct and Indirect Effects of ADHD on Suicide Plans via Mental Health and Substance Problems With Co-Variates Gender and Age.

Variable	Direct effects			Indirect effects			
	Stage 1 *OR* (95% CI)	Stage 2 *OR* (95% CI)	Stage 3 *OR* (95% CI)	Alcohol scores ß (*SE*)	Drug use scores ß (*SE*)	GAD ß (*SE*)	Depression ß (*SE*)
ADHD (none)	3.347*** (2.482–4.514)	3.339*** (2.519–4.586)	2.223*** (1.594–3.099)	.025 (0.031)	.084 (0.028)**	−.011 (0.038)	.359 (0.066)***
Gender (male)	—	0.910 (0.666–1.244)	0.938 (0.669–1.316)	−.011 (0.014)	−.057 (0.018)**	−.005 (0.015)	.039 (0.027)
Age (under 21)	—	1.850*** (1.335–2.565)	1.862** (1.311–2.645)	−.011 (0.014)	.042 (0.020)*	−.001 (0.003)	.001 (0.029)
Alcohol scores	—	—	1.011 (0.984–1.039)	—	—	—	—
Drug use scores	—	—	1.117*** (1.055–1.183)	—	—	—	—
GAD	—	—	0.996 (0.996–1.023)	—	—	—	—
Depression	—	—	1.099*** (1.069–1.129)	—	—	—	—

*Note. OR* = odds ratio; CI = confidence interval; ß = beta coefficient; *SE* = standard error. All significant values are presented in bold.

*** *p* < .001. **p < .01. *p < .05.

**Table 5.** Direct and Indirect Effects of ADHD on Suicide Attempt via Mental Health and Substance Problems With Co-Variates Gender and Age.

Variable	Direct effects			Indirect effects			
	Stage 1 *OR* (95% CI)	Stage 2 *OR* (95% CI)	Stage 3 *OR* (95% CI)	Alcohol scores ß (*SE*)	Drug use scores ß (*SE*)	GAD ß (*SE*)	Depression ß (*SE*)
ADHD (none)	2.652*** (1.782–3.945)	2.694*** (1.810–4.010)	1.753** (1.148–2.678)	.036 (0.041)	.116 (0.035)**	.001 (0.043)	.249 (0.066)***
Gender (male)	—	1.038 (0.676–1.594)	1.154 (0.735–1.811)	−.016 (0.018)	−.079 (0.024)**	−.003 (0.018)	.027 (0.19)
Age (under 21)	—	2.359*** (1.563–3.560)	2.404*** (1.565–3.693)	−.016 (0.019)	.058 (0.026)*	.000 (0.003)	.001 (0.020)
Alcohol scores	—	—	1.016 (0.981–1.052)	—	—	—	—
Drug use scores	—	—	1.165*** (1.093–1.242)	—	—	—	—
GAD	—	—	1.000 (0.970–1.032)	—	—	—	—
Depression	—	—	1.068*** (1.035–1.101)	—	—	—	—

*Note. OR* = odds ratio; CI = confidence interval; ß = beta coefficient; *SE* = standard error. All significant values are presented in bold.

*** *p* < .001. **p < .01. *p < .05.

**Table 6.** Direct and Indirect Effects of ADHD on Self-Harm via Mental Health and Substance Problems With Co-Variates Gender and Age.

Variable	Direct effects			Indirect effects			
	Stage 1 *OR* (95% CI)	Stage 2 *OR* (95% CI)	Stage 3 *OR* (95% CI)	Alcohol scores ß (*SE*)	Drug use scores ß (*SE*)	GAD ß (*SE*)	Depression ß (*SE*)
ADHD (none)	2.886*** (2.153–3.867)	2.901*** (2.163–3.891)	1.964*** (1.420–2.718)	.058 (0.030)	.032 (0.023)	.025 (0.035)	.290 (0.057)***
Gender (male)	—	1.386 (0.999–1.923)	1.393 (0.987–1.966)	−.025 (0.015)	−.022 (0.016)	.010 (0.015)	.031 (0.022)
Age (under 21)	—	1.012 (0.715–1.433)	1.021 (0.710–1.468)	−.026 (0.015)	.016 (0.013)	.002 (0.005)	.001 (0.024)
Alcohol scores	—	—	1.026* (1.001–1.052)	—	—	—	—
Drug use scores	—	—	1.043 (0.984–1.105)	—	—	—	—
GAD	—	—	1.009 (0.984–1.035)	—	—	—	—
Depression	—	—	1.079*** (1.053–1.106)	—	—	—	—

*Note. OR* = odds ratio; CI = confidence interval; ß = beta coefficient; *SE* = standard error. All significant values are presented in bold.

*** *p* < .001. *p < .05.

##### Stage 2

When the covariates gender and age were included in the model the effect of ADHD remained significant, with little change in the odds ratios for any of the suicidal behavior outcome variables or self-harm. Gender was not a significant predictor. Age however predicted suicide ideation (*OR* = 1.594), plan (*OR* = 1.850), and attempt (*OR* = 2.359), with those aged 21 and over more likely to endorse suicidal behavior. Conversely, age was not a significant predictor of self-harm.

##### Stage 3

When the mental health, alcohol, and substance use mediators were included in the final model, the direct pathways between ADHD and the outcome variables which were significant in the previous models remained significant, but the odds reduced considerably as shown in Tables 3 to 6. This would indicate that partial mediation occurred. Age also remained a significant predictor of suicide ideation, plans and attempt but there was little change in the odds ratios. Substance use and depression were significant predictors of suicide ideation, plans and attempts, while alcohol use and depression were direct predictors of self-harm.

##### Indirect effects

Significant indirect effects were revealed for suicide ideation, plans and attempts, with ADHD and the co-variates age and gender having indirect effects via drug use (Tables 3–6). An indirect effect was also revealed via depression for suicidal ideation (ß = .247, *SE* = 0.057, *p* < .0001), plans (ß = .359, *SE* = 0.066, *p* < .001), attempts (ß = .249, *SE* = 0.066, *p* < .001), and self-harm (ß = .290, *SE* = 0.057, *p* < .001).

##### A paths

A number of significant direct effects of ADHD and the covariates age and gender on mental health and substance problems were revealed (*a* paths). Alcohol problems were predicted by age (ß = −1.016, *SE* = 0.344, *p* < .001), gender (ß = −.967, *SE* = 0.319, *p* < .001), and ADHD (ß = 2.243, *SE* = 0.379, *p* < .001), with males and those under 21 more likely to have problems. Drug use was also predicted by age (ß = .382, *SE* = 0.153, *p* < .05), gender (ß = .515, *SE* = 0.134, *p* < .001), and ADHD (ß = .757, *SE* = 0.163, *p* < .001) with males and those 21 and above more likely to have problems. Anxiety was predicted by gender (ß = 1.113, *SE* = 0.353, *p* < .01) and ADHD (ß = 2.726, *SE* = 0.367, *p* < .001), with females at a greater risk. While depression was predicted by having ADHD (ß = 3.803, *SE* = 0.330, *p* < .001).

## Discussion

Among this large sample of NI and ROI college students, a very high prevalence of ADHD was observed (27.2%). Furthermore, high rates of SI (44.5%), plans (26.6%), attempts (13.4%), and NSSI (23.4%) were revealed among those with ADHD. The prevalence of suicidal behaviors and NSSI was significantly higher when compared to students without ADHD. Additionally, even after adjusting for comorbid disorders, substance and alcohol use, significant direct associations between ADHD and suicidality and ADHD and NSSI remained. Depression was found to partially mediate the association between ADHD and suicide behaviors and NSSI. Lastly, ADHD and the co-variates age and gender also had indirect effects on suicidal behaviors via substance use.

The present study found that the prevalence of ADHD among undergraduate students was considerably higher (27.2%) than previously reported (2%–8%) by DuPaul et al. (2009). However, the current prevalence is similar to that reported in an Australian college sample in a recent cross-national study (27.7%) (Mak et al., 2021). This emerging research indicates a high incidence of ADHD among college student samples, with rates ranging from 16% to 25% (Bakhshani et al., 2012; Mak et al., 2021) and implies that the prevalence of ADHD may be higher in college students than in the general population, with authors suggesting high rates of ADHD among college students to be a global phenomenon (Mak et al., 2021). Therefore, it may be that ADHD is more common in college students than earlier reports suggest. Nevertheless, literature examining the international prevalence of ADHD among college students remains scarce, therefore further research is required.

As a screening tool the ASRS-v1.1 is designed to include room for false positives in order to decrease the potential for false negatives. Furthermore, although the ASRS-v1.1 is a well-validated, reliable screening tool, in participants presenting with mood and substance disorders, the sensitivity, specificity, and classification accuracy of the ASRS-v1.1 is reduced. Subsequently, the likelihood of false positives increases, due to overlapping symptomology (Dunlop et al., 2018). As mood and substance use problems were prevalent within the current sample, this may have contributed to the higher ADHD prevalence rates recorded.

The current findings are consistent with previous research that has highlighted students with ADHD to be at heightened risk of suicidality (Eddy et al., 2020; Van Eck et al., 2015; Zhong et al., 2021). The present study found that students presenting with ADHD had more than double the rates of SI and thrice the rates of suicide plans. Additionally, the prevalence of suicide attempts and NSSI were almost two and a half times greater amongst students with ADHD. These rates of suicide behaviors were substantially higher than global prevalence rates found among the general student population (Mortier et al., 2018). However, in comparison, those with ADHD in the general adult population experience a five-fold increased risk of suicidality, therefore, educational attainment may act as a protective factor for those with ADHD (Fitzgerald et al., 2019).

The high rate of suicidal behaviors raises concern as such can adversely impact academic outcomes, retention, life satisfaction, and cause considerable psychological distress (Eskin et al., 2016; Mortier et al., 2018). Furthermore, lifetime suicide plans and attempts are some of the strongest predictors of future attempts and suicide completion (Nock et al., 2013). The current findings suggest that students with ADHD present as an at-risk population upon entry into college. Thus, the need for early screening and intervention is imperative to reduce negative outcomes within this vulnerable group.

In accordance with previous literature, current findings demonstrate that mediating variables only partially accounted for the association between ADHD and suicide behaviors (Chen et al., 2019; Ljung et al., 2014). ADHD remained a significant predictor of suicidal behaviors even after adjusting for co-morbid disorders and alcohol and substance use, albeit to a lesser extent. This supports the hypothesis that the symptoms of ADHD, such as impulsiveness and inattention may act as independent risk factors for SI and attempts (Furczyk & Thome, 2014). Previous authors have hypothesized that attentional impairment and impulsivity serve as precursors to rapid and risky decision-making, predisposing those with ADHD to SI and suicide attempts when experiencing emotional distress (Furczyk & Thome, 2014). Furthermore, emerging research has reported associations between poor executive function, as seen in those with ADHD, and increased risk of suicidality (Saffer & Klonsky, 2017). Although results in this area are mixed, many of the studies which have reported no direct effect of ADHD on suicide behaviors have relied on smaller samples which may have reduced the likelihood of detecting a direct effect (Van Eck et al., 2015; Zhong et al., 2021).

Another important finding from the current study is the direct association between ADHD and suicide plans as previous research has indicated contrasting findings (Eddy et al., 2020). The impulsivity associated with ADHD often results in difficulties with premeditation and one’s ability to consider the long-term consequences of behaviors. Therefore, it was predicted that there would be no direct association between ADHD and suicide plans. This finding raises questions regarding theories proposing impulsivity to be the key determinant of suicide risk in those with ADHD. Instead, other core characteristics such as deficits in emotional regulation, preferences for emotion-focused and avoidance-escape coping strategies may play greater contributing roles (Van Eck et al., 2015; Young, 2005). Alternatively, this contrasting finding may have arisen due to the inclusion of only one question assessing the presence of lifetime suicide plans, “Did you ever think about how you might kill yourself (e.g., taking pills, shooting yourself) or work out a plan of how to kill yourself?.” Students may have interpreted suicidal ideation alluding to method as constituting a significant plan. In future, additional questions regarding suicide ideation and significant plan may be included to better distinguish between the two. Additionally, further research determining which specific components of ADHD infer suicide risk is required.

The influence of depression in the context of ADHD may place individuals at high risk for SI, plans, attempts, and NSSI. This finding is in concordance with findings presented by Van Eck et al. (2015), Arsandaux et al. (2021), and Zhong et al. (2021). Furthermore, the current study demonstrates the indirect role that substance use plays in the association between ADHD and SI, plans, and attempts. The results support the theory that ADHD indirectly increases the risk of suicide via commonly occurring co-morbid disorders (Furczyk & Thome, 2014). Chronic impulsivity and inattention can cause severe social and functional impairment in those with ADHD thus significantly increasing susceptibility toward mood and anxiety disorders (Van Eck et al., 2015). Additionally, impulsivity in tandem with high rates of psychopathology and challenges experienced due to college transition may increase the risk of problematic substance use as a means to escape short-term emotional distress (Young, 2005). Consistent with our findings, young, adult males with ADHD are more likely partake in problematic substance use (Sobanski et al., 2007). In turn, depression and substance use have been frequently and strongly associated with increased suicide risk among young people (Pompili et al., 2012; Wang et al., 2019).

Interestingly, unlike previous studies, anxiety did not mediate the relationship between ADHD and suicide behaviors (Van Eck et al., 2015; Zhong et al., 2021). Indeed, research has indicated that anxiety increases as students’ progress through university due to greater academic demands (Bewick et al., 2010). Therefore, it may be later in the college experience that anxiety begins to play a significant mediating role. For example, although Van Eck et al. (2015) found anxiety to be a significant mediator over half of their sample consisted of students not in their first year. Therefore, this may suggest that different mediators have differing effects depending on the stage of college progression.

### Limitations

Although the current study has its strengths, some limitations should be considered. For example, ADHD was screened for using the ASRS-v1.1 alone. While the ASRS-v1.1 is a well-validated, reliable screening tool, a thorough clinical assessment is recommended with historical developmental corroboration. This is to reduce bias and confirm that participants meet the diagnostic criteria for ADHD and other conditions can be excluded.

Additionally, this study utilized data from a cohort at one timepoint only. As students, particularly those with ADHD, face several stressors and challenges as they progress through college, it may be helpful to extend the current findings by utilizing longitudinal data. Plans are in place to conduct follow up surveys with this cohort. Future research could examine whether the prevalence of suicide behaviors change as students with ADHD progress through university and monitor progression in suicidal behaviors. Additionally, it may be beneficial to examine if the extent to which mediators influence the association between ADHD and suicidality changes over time.

## Conclusion

Despite the limitations, the present study has enhanced our understanding of the relationship between ADHD, suicide behaviors and NSSI, as well as the underlying mediating mechanisms. A key strength of the current study is the utilization of a large sample and the inclusion of measures for suicide ideation, plan, attempt, and NSSI. Many previous studies rely on smaller samples and explore SI exclusively (Van Eck et al., 2015; Zhong et al., 2021). Furthermore, to our current knowledge, substance and alcohol use have not been investigated as mediating factors, while anxiety has not been investigated extensively, despite high prevalence rates within this population (Anker et al., 2018). Lastly, unique contributions have been made in terms of establishing the prevalence of suicide behaviors among students with ADHD in NI and ROI undergraduate populations.

Many of the interventions available to students with ADHD focus on improving organizational, planning, and time-management skills to alleviate academic impairment. However, due to the high prevalence of suicidal behaviors and NSSI and considering the mediating effects of depression and substance use, it may be beneficial to integrate evidence-based mental health interventions into these pre-existing support frameworks. For example, preliminary findings demonstrate that group Cognitive Behavioral Therapy programs show promise in not only reducing symptoms of impulsivity and inattention but in reducing depression and substance use in students with ADHD (Anastopoulos et al., 2021; Van der Oord et al., 2020). Moreover, the present study implies that many pupils are leaving secondary education presenting with ADHD symptomology and high risk for suicidality. Therefore, it may be beneficial to consider the development of similar, cost effective, evidence-based transitionary support programs aimed at pupils wishing to attend college.

The current study is beneficial in providing baseline information about the high prevalence of ADHD and accompanying suicide risk in students upon their entry into college. Additionally, as depression and substance use are modifiable risk factors, appropriate strategies and interventions can be put in place to reduce suicide risk among this vulnerable population. Campus wellbeing services may consider screening students for symptoms of ADHD and awareness could be raised about risks presented by this subgroup. Moreover, research like this may help inform the development of more targeted mental health and suicide prevention campaigns, strategies, and interventions aimed specifically at students with ADHD.
